# Supplementary material for: Divergence in a eukaryotic transcription factor’s co-TF dependence involves multiple intrinsically disordered regions
Source: Nat Commun. 2025 Jun 18;16:5340. doi: 10.1038/s41467-025-59244-w (PMC12177071; doi:10.1038/s41467-025-59244-w)
Supplement: Supplementary file 3 — Description of Additional Supplementary Files [file 41467_2025_59244_MOESM3_ESM.pdf]

Description of Additional Supplementary Files

File Name: Supplementary Data 1
Description: Plasmids used in this study
