# Supplementary material for: Divergence in a eukaryotic transcription factor’s co-TF dependence involves multiple intrinsically disordered regions
Source: Nat Commun. 2025 Jun 18;16:5340. doi: 10.1038/s41467-025-59244-w (PMC12177071; doi:10.1038/s41467-025-59244-w)
Supplement: Supplementary file 4 — Supplementary Data 1 [file 41467_2025_59244_MOESM4_ESM.pdf]

Supplementary Data 1. Plasmids used in this study

ID	Plasmid	Derived	Source	Comment
pH246	pGAD-C3		PMID:8978031, gift from Fassler lab	Yeast two-hybrid
pH247	pGBD-C3		PMID:8978031, gift from Fassler lab	Yeast one-hybrid
pH343	pRS306-GAL1pr-mCherry	pRS306	this study	Yeast one-hybrid
pH346	pRS314-GAL4pr-Gal4	pRS314	this study	Yeast one-hybrid
pH349	pGBD-Gal4DBD-ScE1	pH247	this study	Yeast one-hybrid
pH350	pGBD-Gal4DBD-CgE1	pH247	this study	Yeast one-hybrid
pH351	pGBD-Gal4DBD-ScE1-ScAD	pH247	this study	Yeast one-hybrid
pH352	pGBD-Gal4DBD-CgE1-CgAD	pH247	this study	Yeast one-hybrid
pH362	pGBD-Gal4DBD-CgAD	pH247	this study	Yeast one-hybrid
pH363	pGBD-Gal4DBD-ScAD	pH247	this study	Yeast one-hybrid
pH377	pGBD-Gal4DBD-CgE2	pH247	this study	Yeast one-hybrid
pH378	pGBD-Gal4DBD-ScE2	pH247	this study	Yeast one-hybrid
pH379	pGBD-Gal4DBD-ScAD.9aa	pH247	this study	Yeast one-hybrid
pH380	pGBD-Gal4DBD-CgAD.2	pH247	this study	Yeast one-hybrid
pH386	pGBD-Gal4DBD-CgE2.9aa	pH247	this study	Yeast one-hybrid
pH387	pGBD-Gal4DBD-CgAD.1	pH247	this study	Yeast one-hybrid
pH388	pGBD-Gal4DBD-CgE1-ScAD.9aa	pH247	this study	Yeast one-hybrid
pH389	pGBD-Gal4DBD-ScAD.9aa-CgE1	pH247	this study	Yeast one-hybrid
pH390	pGBD-Gal4DBD-ScAD.9aa-CgE2.9aa	pH247	this study	Yeast one-hybrid
pH391	pGBD-Gal4DBD-CgE2.9aa-ScAD.9aa	pH247	this study	Yeast one-hybrid
pH434	pGBD-Gal4DBD-ScPho2AD	pH247	this study	Yeast one-hybrid
pH394	pGAD-ScPho4ΔDBD	pH246	this study	Yeast two-hybrid
pH395	pGBD-Pho2Pho4int	pH247	this study	Yeast two-hybrid
pH396	pGAD-CgPho4ΔDBD	pH246	this study	Yeast two-hybrid
pH050	pET-11a-ScPho4 DBD-6xHis	pET11a	this study	BLI
pH051	pET-11a-CgPho4 DBD-6xHis	pET11a	this study	BLI
bH404	pET-11a-GST-CgPho4	pET11a	this study	PBM
pH073	bRA89, Cas9, CEN/ARS, HygR		PMID: 28405019 gift from Malkova lab	CRISPR
pH173	ScPHO4pr, mNeon, CEN/ARS, LEU2	pRS315	this study	Chimera backbone
pH188	Cg(1-533)	pH173	this study	Chimera
pH194	Sc(1-312)	pH173	this study	Chimera

pH209	Cg(1-112) Sc(100-176) Cg(283-533)	pH173	this study	Chimera
pH210	Cg(1-458) Sc(243-312)	pH173	this study	Chimera
pH211	Cg(1-282) Sc(177-242) Cg(459-533)	pH173	this study	Chimera
pH212	Sc(1-42) Cg(45-533)	pH173	this study	Chimera
pH213	Cg(1-44) Sc(43-99) Cg(113-533)	pH173	this study	Chimera
pH215	Sc(1-99) Cg(113-533)	pH173	this study	Chimera
pH216	Sc(1-42) Cg(45-458) Sc(243-312)	pH173	this study	Chimera
pH217	Cg(1-44) Sc(43-176) Cg(283-533)	pH173	this study	Chimera
pH218	Cg(1-112) Sc(100-242) Cg(459-533)	pH173	this study	Chimera
pH219	Cg(1-282) Sc(177-312)	pH173	this study	Chimera
pH220	Sc(1-42) Cg(45-112) Sc(100-176) Cg(283-533)	pH173	this study	Chimera
pH221	Cg(1-44) Sc(43-99) Cg(113-282) Sc(177-312)	pH173	this study	Chimera
pH222	Sc(1-42) Cg(45-282) Sc(177-312)	pH173	this study	Chimera
pH223	Cg(1-44) Sc(43-99) Cg(113-282) Sc(177-242) Cg(459-533)	pH173	this study	Chimera
pH224	Sc(1-42) Cg(45-112) Sc(100-176) Cg(283-458) Sc(243-312)	pH173	this study	Chimera
pH227	Sc(1-176) Cg(283-533)	pH173	this study	Chimera
pH229	Sc(1-176) Cg(283-458) Sc(243-312)	pH173	this study	Chimera
pH230	Sc(1-99) Cg(113-282) Sc(177-312)	pH173	this study	Chimera
pH231	Cg(1-44) Sc(43-312)	pH173	this study	Chimera
pH232	Sc(1-42) Cg(45-112) Sc(100-312)	pH173	this study	Chimera
pH233	Sc(1-242) Cg(459-533)	pH173	this study	Chimera
pH234	Cg(1-112) Sc(100-312)	pH173	this study	Chimera
pH235	Sc(1-99) Cg(113-458) Sc(243-312)	pH173	this study	Chimera
pH236	Cg(1-76) Sc(71-99) Cg(113-282) Sc(177-312)	pH173	this study	Chimera
pH237	Cg(1-44) Sc(43-70) Cg(77-282) Sc(177-312)	pH173	this study	Chimera
pH239	Sc(1-99) Cg(113-282) Sc(177-242) Cg(459-533)	pH173	this study	Chimera
pH240	Cg(1-112) Sc(100-176) Cg(283-458) Sc(243-312)	pH173	this study	Chimera

pH241	Cg(1-44) Sc(43-242) Cg(459-533)	pH173	this study	Chimera
pH250	Cg(1-44) Sc(43-176) Cg(283-458) Sc(243-312)	pH173	this study	Chimera
pH251	Sc(1-42) Cg(45-282) Sc(177-242) Cg(459-533)	pH173	this study	Chimera
pH252	Sc(1-42) Cg(45-112) Sc(100-242) Cg(459-533)	pH173	this study	Chimera
pH253	Cg(1-44) Sc(43-99) Cg(113-458) Sc(243-312)	pH173	this study	Chimera
pH254	Cg(1-44) Sc(43-176) Cg(283-327) Sc(205-242) Cg(459-533)	pH173	this study	Chimera
pH255	Cg(1-44) Sc(43-204) Cg(328-533)	pH173	this study	Chimera
pH256	Cg(1-44) Sc(43-220) Cg(377-533)	pH173	this study	Chimera
pH257	Cg(1-282) Sc(177-204) Cg(328-533)	pH173	this study	Chimera
pH258	Cg(1-327) Sc(205-242) Cg(459-533)	pH173	this study	Chimera
pH265	Sc(1-204) Cg(328-533)	pH173	this study	Chimera
pH266	Sc(1-153) Cg(250-533)	pH173	this study	Chimera
pH276	Sc(1-42) Cg(45-282) Sc(177-204) Cg(328-533)	pH173	this study	Chimera
pH277	Cg(1-44) Sc(43-99) Cg(113-282) Sc(177-204) Cg(328-533)	pH173	this study	Chimera
pH278	Sc(1-204) Cg(328-458) Sc(243-312)	pH173	this study	Chimera
pH279	Cg(1-44) Sc(43-204) Cg(328-458) Sc(243-312)	pH173	this study	Chimera
pH294	Cg(1-470) Sc(251-312)	pH173	this study	Chimera
pH301	Sc(1-247) Cg(464-533)	pH173	this study	Chimera
pH326	Sc(1-156) Cg(253-533)	pH173	this study	Chimera
pH327	Cg(1-252) Sc(157-312)	pH173	this study	Chimera
pH328	Sc(1-42) Cg(45-252) Sc(157-312)	pH173	this study	Chimera
pH329	Cg(1-44) Sc(43-247) Cg(464-533)	pH173	this study	Chimera
pH330	Cg(1-44) Sc(43-156) Cg(253-463) Sc(251-312)	pH173	this study	Chimera
pH331	Cg(1-44) Sc(43-156) Cg(253-533)	pH173	this study	Chimera
pH332	Sc(1-42) Cg(45-463) Sc(251-312)	pH173	this study	Chimera
pH334	Sc(1-42) Cg(45-252) Sc(157-247) Cg(464-533)	pH173	this study	Chimera

pH436	Cg(1-112) Sc(100-176) Cg(283-458) Sc(177-312)	pH173	this study	Chimera
pH438	Sc(1-176) Cg(283-458) Sc(177-312)	pH173	this study	Chimera
pH440	Cg(1-44) Sc(43-99) Cg(113-458) Sc(177-312)	pH173	this study	Chimera
pH441	Sc(1-42) Cg(45-458) Sc(177-312)	pH173	this study	Chimera
pH442	Sc(1-99) Cg(113-458) Sc(177-312)	pH173	this study	Chimera
