# Supplementary material for: Divergence in a eukaryotic transcription factor’s co-TF dependence involves multiple intrinsically disordered regions
Source: Nat Commun. 2025 Jun 18;16:5340. doi: 10.1038/s41467-025-59244-w (PMC12177071; doi:10.1038/s41467-025-59244-w)
Supplement: Supplementary file 5 — Reporting Summary [file 41467_2025_59244_MOESM5_ESM.pdf]

nature portfolio

Corresponding author(s): Bin Z. He

Last updated by author(s): Feb 14, 2025

Reporting Summary

Nature Portfolio wishes to improve the reproducibility of the work that we publish. This form provides structure for consistency and transparency in reporting. For further information on Nature Portfolio policies, see our Editorial Policies and the Editorial Policy Checklist.

Statistics

For all statistical analyses, confirm that the following items are present in the figure legend, table legend, main text, or Methods section.

n/a	Confirmed	
☐	☒	The exact sample size (*n*) for each experimental group/condition, given as a discrete number and unit of measurement
☐	☒	A statement on whether measurements were taken from distinct samples or whether the same sample was measured repeatedly
☐	☒	The statistical test(s) used AND whether they are one- or two-sided<br>*Only common tests should be described solely by name; describe more complex techniques in the Methods section.*
☒	☐	A description of all covariates tested
☐	☒	A description of any assumptions or corrections, such as tests of normality and adjustment for multiple comparisons
☐	☒	A full description of the statistical parameters including central tendency (e.g. means) or other basic estimates (e.g. regression coefficient) AND variation (e.g. standard deviation) or associated estimates of uncertainty (e.g. confidence intervals)
☐	☒	For null hypothesis testing, the test statistic (e.g. *F*, *t*, *r*) with confidence intervals, effect sizes, degrees of freedom and *P* value noted<br>*Give P values as exact values whenever suitable.*
☒	☐	For Bayesian analysis, information on the choice of priors and Markov chain Monte Carlo settings
☒	☐	For hierarchical and complex designs, identification of the appropriate level for tests and full reporting of outcomes
☐	☒	Estimates of effect sizes (e.g. Cohen's *d*, Pearson's *r*), indicating how they were calculated

Our web collection on statistics for biologists contains articles on many of the points above.

Software and code

Policy information about availability of computer code

Data collection	ForteBio Data Analysis (v11) for Biolayer Interferometry data collection; GenePix Pro (v 7.0) for scanning the microarray used in the universal and genome context Protein Binding Microarray; ImageStudio by Li-COR for EMSA image acquisition. Attune NxT software v3.1 for flow cytometry data acquisition.
Data analysis	ForteBio Data Analysis (v11) for Biolayer Interferometry data analysis; ImageJ (v1.53) was used to quantify Pho4 nuclear concentration and fraction; all other analyses were performed using custom R and Python scripts. All custom scripts are made available through out public GitHub repository https://doi.org/10.5281/zenodo.14501732

For manuscripts utilizing custom algorithms or software that are central to the research but not yet described in published literature, software must be made available to editors and reviewers. We strongly encourage code deposition in a community repository (e.g. GitHub). See the Nature Portfolio guidelines for submitting code & software for further information.

Data

Policy information about availability of data

All manuscripts must include a data availability statement. This statement should provide the following information, where applicable:

- Accession codes, unique identifiers, or web links for publicly available datasets
- A description of any restrictions on data availability
- For clinical datasets or third party data, please ensure that the statement adheres to our policy

Source data for figures in this paper are available at https://doi.org/10.5281/zenodo.14501732. Raw microscopy images for quantifying Pho4 nuclear concentration

are available from the corresponding author upon request. No restrictions apply to any of the data generated in this study.

Research involving human participants, their data, or biological material

Policy information about studies with human participants or human data. See also policy information about sex, gender (identity/presentation), and sexual orientation and race, ethnicity and racism.

Reporting on sex and gender	NA
Reporting on race, ethnicity, or other socially relevant groupings	NA
Population characteristics	NA
Recruitment	NA
Ethics oversight	NA

Note that full information on the approval of the study protocol must also be provided in the manuscript.

Field-specific reporting

Please select the one below that is the best fit for your research. If you are not sure, read the appropriate sections before making your selection.

☒ Life sciences ☐ Behavioural & social sciences ☐ Ecological, evolutionary & environmental sciences

For a reference copy of the document with all sections, see nature.com/documents/nr-reporting-summary-flat.pdf

Life sciences study design

All studies must disclose on these points even when the disclosure is negative.

Sample size	No sample size calculation was used. A minimum of three biological replicates were used in all experiments (n>=5 for most experiments). This decision was based on experience with the experiment types in our lab and also conventions in the field.
Data exclusions	In our flow cytometry data acquisition, we placed a number of common control samples in each run to control for data consistency. In post analysis, when the common control samples show abnormally high or low activity, defined as >50% above or below the mean of the rest of the data, the entire plate is excluded from further analyses. The same plate is rerun.
Replication	For yeast strains, we generate and store at least two independent transformants. Whenever possible, we test two or more transformants to determine if two transformants exhibit different behaviors, we test two more transformants to arrive at a conclusion. The results presented in the main article have all been confirmed this way.
Randomization	In our flow cytometry experiments, 96-well plates were used and the samples were randomized on the plate. In particular, we place common control samples on fixed positions to ensure consistency across plates. The same genotype is run on at least two plates, where its position is intentionally altered to ensure no positional effects obscures the observed values.
Blinding	When quantifying the nuclear fractions and concentrations of the Pho4 constructs, the images were taken and the file names were indexed by date and number only. The genotype of the cells in the images were blinded. The researcher quantifies the image, and a different researcher matched the values to the genotypes.

Reporting for specific materials, systems and methods

We require information from authors about some types of materials, experimental systems and methods used in many studies. Here, indicate whether each material, system or method listed is relevant to your study. If you are not sure if a list item applies to your research, read the appropriate section before selecting a response.

Materials & experimental systems

n/a	Involved in the study	
☒	☐	Antibodies
☒	☐	Eukaryotic cell lines
☒	☐	Palaeontology and archaeology
☒	☐	Animals and other organisms
☒	☐	Clinical data
☒	☐	Dual use research of concern
☒	☐	Plants

Methods

n/a	Involved in the study	
☒	☐	ChIP-seq
☐	☒	Flow cytometry
☒	☐	MRI-based neuroimaging

Plants

Seed stocks: NA

Novel plant genotypes: NA

Authentication: NA

Flow Cytometry

Plots

Confirm that:

- [x] The axis labels state the marker and fluorochrome used (e.g. CD4-FITC).
- [x] The axis scales are clearly visible. Include numbers along axes only for bottom left plot of group (a 'group' is an analysis of identical markers).
- [x] All plots are contour plots with outliers or pseudocolor plots.
- [x] A numerical value for number of cells or percentage (with statistics) is provided.

Methodology

Sample preparation: yeast cells were grown overnight, diluted in the morning and regrown to mid log phase before being measured

Instrument: Attune NxT

Software: Attune NxT software v3.1

Cell population abundance: a total of 30,000 events were collected; after gating, the relevant population's abundance is on average 10,000. samples with fewer than 5,000 events are repeated.

Gating strategy: a "cell" gate was used to exclude non-cell events based on FSC.H and SSC.H; a second "singel cell" gate was then used on the selected "cell" population to exclude doublets/multilets based on FSC.H and FSC.W

- [x] Tick this box to confirm that a figure exemplifying the gating strategy is provided in the Supplementary Information.
